# A perceptual sound space for auditory displays based on sung-vowel synthesis

**DOI:** 10.1038/s41598-022-23736-2

**Published:** 2022-11-12

**Authors:** Davide Rocchesso, Salvatore Andolina, Giacomo Ilardo, Salvatore Danilo Palumbo, Ylenia Galluzzo, Mario Randazzo

**Affiliations:** Department of Mathematics and Computer Science, University of Palermo, Palermo, 90123 Italy

**Keywords:** Computer science, Electrical and electronic engineering

## Abstract

When designing displays for the human senses, perceptual spaces are of great importance to give intuitive access to physical attributes. Similar to how perceptual spaces based on hue, saturation, and lightness were constructed for visual color, research has explored perceptual spaces for sounds of a given timbral family based on timbre, brightness, and pitch. To promote an embodied approach to the design of auditory displays, we introduce the Vowel–Type–Pitch (VTP) space, a cylindrical sound space based on human sung vowels, whose timbres can be synthesized by the composition of acoustic formants and can be categorically labeled. Vowels are arranged along the circular dimension, while voice type and pitch of the vowel correspond to the remaining two axes of the cylindrical VTP space. The decoupling and perceptual effectiveness of the three dimensions of the VTP space are tested through a vowel labeling experiment, whose results are visualized as maps on circular slices of the VTP cylinder. We discuss implications for the design of auditory and multi-sensory displays that account for human perceptual capabilities.

## Introduction

Information living in a data space can be made accessible to the human senses by employing perceptualization processes^1^. The relevant data dimensions, possibly extracted with dimensionality reduction methods, can be mapped to the coordinates of some space that makes sense to humans, or a perceptual space. Points within such space would become perceivable stimuli if a display device is capable to synthesize them, and the space itself becomes the control playground for the human data analyst or the display designer.

Perceptual spaces for displays (e.g., a Hue-Saturation-Lightness space for color, HSL) have some characteristics that make them preferable to the direct control of display-device parameters (e.g., Red-Green-Blue values): Intuitive addressability (specification in perceptual terms); Uniformity (proximity of points implies proximity of sensations); Independence of the control dimensions. Although these properties are achieved only approximately in practice, a given display device can be characterized in terms of a volume (gamut) in the given perceptual space and controlled through a perceptual interface^2^.

Research in sonification aims at making data properties and relations audible, through the “acoustic representation of informational data for relational non-linguistic interpretation by listeners”^3^. The construction of perceptual spaces in this domain is often a synonym of parameter mapping^4^, “which represents changes in some data dimension with changes in an acoustic dimension to produce a sonification”, where “the dimensionality of the data must be constrained such that a perceivable display is feasible”^5^.

Ideally, the dimensions of a perceptual sound space for sonification should be interpretable, linear, and possibly orthogonal^6^. The presence of a clear coordinate origin point is a requirement for multidimensional spaces with bipolar axes, but we may have a very usable perceptual space arranged in the form of a cylinder with a well-defined axis, as in the HSL space for color, where the radial dimension grows out of the axis, and the circular dimension has only a conventional zero^7^.

The vast knowledge in psychoacoustics provides several sound features that can be extracted from sound analysis and used to drive sound synthesis. For example, a three-dimensional sound space with bipolar axes has been constructed^6^ based on auditory qualities such as chroma, brightness, roughness, fullness, and beats, and used to control a Shepard-tone synthesizer in a variety of sonification contexts^8^. Such sonifications, being grounded on perceptual qualities, require only limited training and can achieve good precision. However, the generated streams are cognitively distant from everyday experiences and soundscapes. It has indeed been argued that constructing “intentional inexistent objects” out of pitch or brightness variations fails to account for the embodied aspects of sound perception and production, thus limiting their effectiveness^3^. On the other hand, when embodied cognition guides sonic information design, often the results are compelling, robust to context variations, and readily understood^9,10^. This suggests choosing sounds that themselves have proven to have familiar embodied associations for a listener. In this respect, there is one category of sounds that everyone becomes familiar with even before birth, that is the human voice.

Decades of experience and efforts in the field of auditory display suggest that effective sonification should be sought by research and experimentation along two shifted paths^11^: An artistic shift that would prioritize usefulness in design, possibly sacrificing veridicality; An empirical shift where: (1) Design efforts should focus on those perception dimensions where audition performs well and individual differences are smallest; (2) The perceptual interactions between simple acoustic dimensions, like pitch and loudness, are minimized; (3) The target listener is not the music-educated analytical listener. Non-speech voice synthesis and a perceptual space based on the principal dimensions of the human voice have the potential of meeting the expectation for effective sonifications^12^. The embodied advantage of voice-like sound synthesis for sonification is both in terms of perception (humans are good at detecting differences and nuances) and in terms of action or communication, as vocal imitations are the embodied means of sonic sketching^13^ and voice-like sounds can be readily imitated. This also points to a possible drawback of voice-based sonification, which is its possible interference with speech communication. However, speech uses only a subset of possible vocal sounds and, as it happens to animal communication in complex environments, any sonification should be designed within an acoustic and articulatory niche that minimizes communication interferences. Some application examples have already shown the effectiveness of an embodied approach to sonification and audio feedback design, using non-speech voice synthesis^14–17^ or the nonverbal prosodic content of utterances^18,19^.

In the visual domain, research in color perceptual spaces has been extensive and produced spaces where hue and saturation are reported through transformations of chromaticity coordinates, and the lightness dimension is treated as special^1^. Many proposals have been advanced and tested to create correspondences between color patches and stimuli to the other senses, especially in the context of sensory substitution^20^. Proceeding by analogy with color models and their related color specification interfaces, Barrass proposed a perceptual space for sound to be used in auditory displays, using the dimensions of Timbre, Brightness, and Pitch (TBP)^7,21,22^. The problem in defining and using such auditory perceptual spaces is the vagueness and vastness of the concept of timbre so that the space can be precisely defined and constructed only within a given timbral family, a sort of pre-defined orchestra the sound designer can work with. In practical implementations of TBP, one has to start from a set of audio samples of different timbres at different pitches and apply audio transposition and timbre morphing to steer a trajectory in the three-dimensional sound gamut.

Among the many possible timbral families, we have seen that the most natural choice for embodied sound design and interaction is the family of human voices. In particular, in this work, we restrict our attention to the space of vowels, as parametric sound synthesis models of vowel sounds are readily available, vowels are founding elements of acoustic communication between humans, and a small set of vowels is relatively culture and language independent. In particular, we focus on sung vowels, which are characterized by the relative steadiness of pitch when singing a given note. Sung vowels are likely to emerge in acoustically cluttered environments, as they do within a music orchestra, without interfering much with speech communication. In Western classical music, voices are grouped according to voice types (from bass to soprano), each characterized by a pitch range (*tessitura*) in a small set of production modes (vocal registers).

From the literature on sound and music computing several sound models can be borrowed to describe and generate sung vowel sounds^23^, and some implementations have been proposed and made available for the purpose of information sonification^24^. For our realization, we chose the time-domain formant-wave-function (*Fonction d’Onde Formantique*—FOF) synthesis^25^, for its efficiency, simplicity, and intuitiveness of parametric control. FOF synthesis is available in a variety of languages and environments, including the versatile Faust real-time signal processing language^26^.

We propose the three-dimensional perceptual Vowel-Type-Pitch (VTP) sound space based on categorizable Vowels, ordinally-arranged voice Types, and an interval scale of Pitches. The resulting cylindrical volume is mapped to the parameters of a FOF synthesizer, thus making it possible to continuously change vowel and pitch, with no need for audio signal processing for morphing and transposition, and no memory needed to store audio samples. To investigate the potential effectiveness of the VTP space in supporting real-world sonification applications through embodied auditory displays, we implemented a vowel synthesizer based on the VTP space in a mobile app.

For testing how robust and consistent the categorization of vowels is across different types and pitches, we conducted a vowel labeling experiment across five voice types and a wide range of pitches. The experimental results are proposed in visual form, to help define a three-dimensional gamut of synthetic sounds for information sonification and auditory display. While vowel recognition has been extensively assessed in the context of the acoustics of the singing voice^27–30^, no study has been previously conducted on a voice synthesizer for its suitability as the engine of a sound information space. The proposed sound space, its realization based on the sung-vowel synthesis, and the results of the study will be beneficial to information designers who are willing to use sound for data representation. Representation of ordinal or interval data is possible via two of the space dimensions, and sonic palettes can be designed, made of discrete points of the space that can be consistently named. Moreover, the proposed vowel space is suitable for continuous sonic interaction^31^, as human movements can be mapped into trajectories within the space, and made audible as vocal gestures.

## Background

### Information sound spaces

The concept of Information Sound Spaces (ISS) was introduced by Barrass^7,21,22^ to denote a special kind of cognitive artifact for auditory design. In analogy to the HSL color space^1^, Barrass proposed a cylindrical perceptual space for sounds with the following three dimensions:

- *Timbre* Attribute which enables sound object identification. Nominal scale for categorical association (analogue to Hue).
- *Brightness* Attribute according to which sounds can be ordered from dull to sharp. Ratio scale with original zero, that is the pure tone (analogue to Saturation).
- *Pitch* Attribute according to which sounds can be ordered from low to high. Interval scale (analogue to Lightness).

These definitions are somehow simplified, as pitch and timbre are themselves multi-dimensional, and brightness is a dimension of timbre^32,33^.

Pitch is problematic in perceptual mapping because of the aggregating power of the octave: In one sense two notes that are a semitone apart are closer than two notes that are separated by twelve semitones (or an octave), but in another sense, the octave notes are closer and more confusable than the semitone, being in fact coincident in terms of chroma^32^. A zero-pitch can be established by convention (e.g., the A0 piano key), and the corresponding sound may or may not be audible depending on timbre spectral richness, and characteristics of the display device. The absence of a natural zero induces to consider pitch more as interval than ratio scale. Despite its complexity, pitch is an indispensable attribute of sound, in the sense that it is necessary for judgments of perceptual numerosity^34^, it is the strongest sound attribute for auditory stream segregation^35^, and is by far the most used auditory dimension in sonification mappings^4^, where its high resolution and large range are generally appreciated.

An operational definition of brightness can be given through a shelving filter whose lowest possible cutoff frequency is set to the fundamental^21^. Experiments have shown that brightness scaling is possible, or estimates of brightness ratios can be given by humans^36^. Brightness is often described as the perceptual correlate of the spectral centroid or center of mass of the spectral distribution, and a correction to the spectral centroid as a determinant of the pitch has been proposed, through subtraction of the fundamental frequency^37^. At a given pitch, brightness offers a ratio scale with an original zero on the dull axis, where the centroid collapses onto pitch^21^. Pitch and brightness are commonly treated as orthogonal when designing experiments to compare the different dimensions. For example, to measure the ability to retain contours encoded either through pitch or through brightness, stimuli were prepared so that for pitch encoding, the spectral envelope is fixed and fundamental is shifted, and for brightness encoding, the pitch is fixed and the spectral envelope is shifted^38^. Although they have different resolutions, and brightness resolution is pitch-dependent, pitch and brightness are often taken together to sonify points in a two-dimensional space, with a general preference for mappings where pitch is associated with the vertical dimension^39^.

The TBP sound space becomes a proper perceptual sound space as a result of scaling operations and perceptual calibration of the axes, which are dependent on the particular set of exemplars that are chosen for the timbre pedestal. In particular, eight sustained musical-instrument tones, derived from prior timbre studies with multidimensional scaling, were selected by Barrass from their planar projections and arranged around a timbre circle, and their brightness was controlled by adjusting the cutoff frequency of a lowpass shelving filter^7^. In this way, two opposite timbres on the circle have maximal perceptual distance. However, seamless transitions between neighboring timbres are possible only by some form of audio timbre morphing^40^. This would be important to achieve a space where timbre is globally categorical yet locally continuous, similarly to the hue in the HSL color space.

In general, a three-dimensional Information Sound Space should realize a mapping between data and sound that can render category (nominal), order (ordinal), and magnitude of difference (interval or ratio)^22^. The fact that sound, compared to color, has a much higher dimensionality makes the definition of information sound spaces challenging.

### The space of vowels

**Figure 1:**
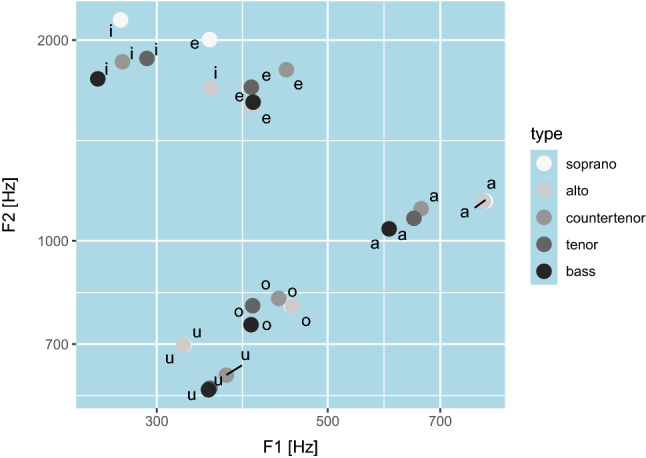
Disposition of the five cardinal vowels /a, e, i, o, u/ on the plane of the lowest two formant frequencies F1 and F2, for five different voice types. The vowel coordinates are commonly used as preset parameters in singing-voice synthesizers^26,45^.

The space of vowels is described by the positions of a few (typically five) formants that characterize any given vowel. The formants are broad resonances of the vocal tract, that act as a filter imposing an amplitude envelope to a spectrally-rich excitation of the vocal folds (phonation, for voiced vowels) or turbulent sources (for unvoiced vowels)^27^. Most of the variance of the vowel space is captured by the lowest two formants, with resonances centered at frequencies F1 and F2, respectively. The space of vowels is locally continuous, but non-linear warping occurs perceptually^41^, so that different areas can be given different labels from a discrete set of vowel names, and the space is globally categorical. It is customary to locate the vowels on the F1–F2 plane, as in Fig. 1. Different languages use different kinds and numbers of vowels, and their discriminability is culture-dependent, but the set /a, e, i, o, u/ (here we use the International Phonetic Alphabet symbol set, as in the literature of singing voice^29^) is found in most languages and, given a voice type, they are well separated in the F1–F2 plane. The combination of tongue backness and lip roundedness^42^ allows to move along a V-like trajectory in the F1–F2 plane, where /i, a, u/ are the corner vowels, maximally distant from each other. The vowels /a, e, i, o, u/ are the five “cardinal vowels” of *bel canto*, where they are used much more extensively than other vowels^29^. For auditory information design, with a set of vowels larger than /a, e, i, o, u/, it is likely that some would be more easily confused by listeners, as they tend to cluster in the space of formant frequencies^21^.

Hermann and Baier were among the first to show how the space of vowels could be used for sonification purposes^14^, limiting pitch to a speech-like range and proposing a transition from unvoiced to voiced vowels to emphasize episodes of deviation from normality. Grond and Hermann realized the first vowel-based synth for the auditory display of mathematical functions, where the function value was mapped to pitch, the second derivative was mapped to brightness, and the first derivative was mapped to the segment /a, e, i/ of the vowel space^15^. The use of the most important voice formants for the purpose of data sonification was also proposed by Ferguson et al.^16^, who judged the resulting tones as perceptually rich yet not overly complex, due to their speech-like character. This sonification was associated with Chernoff’s faces in information visualization^43^, as both kinds of perceptualization rely on the ability of humans to easily recognize human qualities and notice small changes in the represented data items. Both visualization by faces and sonification by vowels can be considered to be forms of embodied information design, as long as data are disguised as human-like objects that are readily perceived. Roddy and Furlong^44^ showed, through a crowdsourcing study, that vowel formant profiles can be associated with embodied attribute schemas such as strong-weak, big-small, and dark-bright, and that the amount of noise in the excitation can modulate the amount of represented tension.

Compared to their use in speech, vowels in singing are used as musical notes, being sustained longer and more steadily, and if we consider the different voice types that can sing vowels, the range of pitches extends over several octaves. Similar to information visualization^1^, the vowels/notes can be thought of as glyphs for auditory scatterplots, as long as one or more quantitative data attributes are mapped in a systematic way to their different auditory properties.

A voice type is characterized by the distribution of formants for each vowel, and by the pitch range it can most comfortably sing. Figure 1 shows where the vowels of each voice type are positioned in the F1-F2 plane. Some vowels, as sung by different voice types, seem to overlap in the F1–F2 plane, but they are actually distinct if represented in a higher-dimensional space, where formants up to the fifth are considered^45^. In operatic singing, voice types are treated as different instruments, each with a suitable repertoire. However, there is also an ordering in voice types due to both the absolute frequency position of the formants and to the pitch ranges they can afford, which makes them suitable to be arranged along the ordinal axis of an information sound space. For example, tenors have higher formant frequencies (see Fig. 1) and can reach higher pitches compared to bass singers. The vowels of operatic singing have other peculiarities as well, such as the clustering of the third, fourth, and fifth formant frequencies to produce the so-called singer’s formant and the fact that sopranos raise the first formant according to pitch, for pitches above $700~{\textrm{Hz}}$^27^.

Sound synthesis models of the singing voice normally keep the pitch, vowel, and type dimensions separate as three independent parameters, thus losing much of the limitations and dimensional interactions found in real singing^23,45^. In this way, however, the control space is more clearly defined and easier to explore, even with combinations of parameters that would not be achievable with human singers.

## The vowel-type-pitch space

For data sonification and auditory displays, we propose an information sound space that can be represented as a cylinder, whose continuous circular dimension can be reduced to categories (Vowel), radial dimension is ordinal (voice Type), and longitudinal dimension is interval (Pitch). The triangle shape, identifiable in the F1-F2 plane of Fig. 1 with corners /a, i, u/, is actually bent into a circular pedestal for the VTP cylinder, similarly to how the triangle in the chromaticity diagram produces the circle in the HSL model of color^46^.

For the categorical dimension of vowels, it makes sense to use the five cardinal vowels, as they are most widely used and recognized in the singing voice. Indeed, the vowel parameter in typical synthesizers is continuous, thus allowing for interpolation between vowels. This is what happened in previous additive or subtractive vowel synthesizers for auditory display^15,24^. For our experimental realization, we used FOF synthesis as implemented in the Faust real-time signal processing language^26^, and adapted it so that the /a, e, i, u, o/ sequence, which describes a closed triangular path in the formant plane (Fig. 1), would correspond to the vowel parameter varying in the range $\left[ 0, 5\right)$. The sequence is implemented as a continuous parametric path, with interpolation between neighboring vowels.

**Figure 2:**
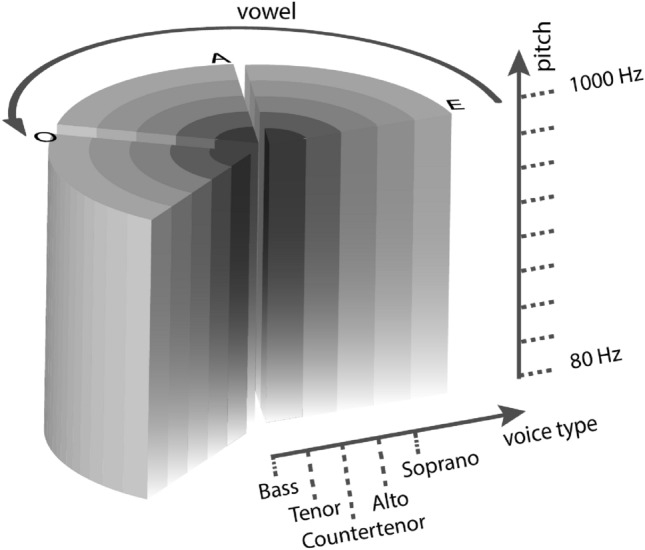
The VTP space. The circular dimension is categorical (vowel), the radial dimension is ordinal (voice type), and the longitudinal dimension is interval (pitch).

In the basic form of the VTP space, depicted in Fig. 2, the voice-type dimension is discretized in a few steps. This happens in operatic singing as well, where the continuum of human voices gets practically and conventionally reduced to a few types. Still, in some cases, it is not easy to discriminate between different voice types, and many singers can perform as different types. For the sake of information sonification, the radial axis type may well be made continuous and monotonically ordinal (actually ratio) by introducing a brightness-control shelving filter, as proposed for TBP spaces^7^. For even smoother variation, voice-type interpolation is also possible. A brightness control based on raising or lowering the amplitude of higher formants was proposed and effectively demonstrated^24^.

### FOF synthesis

Formant-wave-function synthesis^23,25^ provides an additive model of vowels as a bank of resonant filters excited by periodic pulses or, equivalently, as a superposition of sound grains, each characterized by amplitude, oscillation frequency, attack time, and decay time. These parameters can be related to the measured characteristics of vowel formants (central frequency, amplitude, and bandwidth) for different voice types^45^. FOF synthesis allows continuous and seamless interpolation within a cylindrical shell of the space, while the voice-type dimension is discretized in a few steps, corresponding to the formant characteristics of different operatic voices. Pitch can be varied continuously and beyond the normal ranges of operatic voice types, and it does not affect the formant frequencies. The resulting three-dimensional space of parameters pitch, vowel, and type is compact and convex.

### VTP implementation

Faust^26^ is a functional programming language for audio signal processing, that allows translating sets of functional audio streams into code blocks that can be compiled into audio plug-ins or stand-alone apps. A robust and versatile implementation of FOF synthesis was included in the Faust repertoire of synthesis models^26^. This implementation has been exploited to produce a mobile app that can reproduce a random sung vowel in the VTP space. More specifically, the app permits to reproduce the vowel set /a, e, i, u, o/ in a range of pitch (fundamental frequency between $80~{\textrm{Hz}}$ and $1000~{\textrm{Hz}}$) for five voice types:

- *Bass* Male singing voice. It is the lowest vocal range of all voice types, corresponding to a fundamental frequency in the range 82–329 Hz;
- *Tenor* This is the type that most often takes the leading male role in opera. The vocal range for a tenor is about 130–523 Hz;
- *Countertenor* The highest male voice type. The countertenor range is 196–740 Hz;
- *Alto* The alto is the lowest type of female voice. The typical alto range is 174–740 Hz;
- *Soprano* The soprano is the highest female voice type. The vocal range for an operatic soprano is roughly 261–1046 Hz.

The reported ranges are those that human singers can normally afford, but the FOF synthesizer can indeed play each type for any pitch across several octaves. The reported ordering of types, from bass to soprano, corresponds to the five layers, from inner to outer, of the cylindrical space of Fig. 2. Such ordering is a permutation of the one implemented in the FOF synthesizer^47^.

### Object of study

In sonification, the VTP model dimensions may be assigned to data dimensions according to their respective properties. In particular, an interval scale requiring high perceptual resolution would be mapped to the pitch axis, as the just noticeable difference in frequency is as low as 1 Hz in the range of fundamental frequencies between 80 Hz and 500 Hz, and it increases to a few Hertz for higher pitches, so that over 500 frequency steps could be discriminated in the range between 80 Hz and 1000 Hz^48^. A nominal data dimension with a few labels may be mapped to vowels, by exploiting their globally categorical nature, as long as those vowels are reliably labeled. The purpose of this study is to show how and where in the pitch-type space the vowels are consistently labeled. Besides a discrete, nominal mapping, the vowel’s circular dimension also affords continuous trajectories that exploit the locally continuous nature of the vowel space. An ordinal data dimension may be mapped to the voice type radial axis of the VTP model, although in this case we should introduce an explicit radial control of brightness to enforce a perceptual radial order. In real singing, the determination of voice type has more to do with the pitch range a specific type can naturally support, and with the repertoire. In real singing, any voice type is only available in a fraction of the pitch range that is considered here. Types can certainly be distinguished for a given vowel at a given pitch, but their ordering or labeling would be demanding. We insist that control of brightness through filtering should be superimposed on the type axis to impose a perceptual order, as indicated in the original TBP space^49^ as well as in other vocal synthesis models^24^. In the realization used in the present study, however, we did not include a brightness filter, to avoid introducing a confounding variable in the vowel labeling test, that we rather conducted on the FOF synthesis model with no extensions.

## Empirical study of vowel labeling across pitch and voice type

### Research question

We conducted a user study to answer the following research question:

RQ Can humans reliably assign labels (vowel names) to different sectors of the VTP cylindrical sound space, and how robust is such labeling across voice types and in different pitch ranges?

This investigation is necessary to carve a perceptually consistent gamut within the VTP cylinder, where the three dimensions can be all appreciated, and the areas of reliable categorization are highlighted. The task of naming vowels is similar to that of naming colors^1^. In the context of color, naming studies have been shown to be necessary to inform the realization of effective selection, editing, and palette design tools^50^. Similarly, answering the RQ will be beneficial for the construction of sound design tools and auditory displays.

### Ethics

The experimental activities were conducted in compliance with the ethical guidelines of the University of Palermo^51^. The risks were assessed and considered minimal, with no induced distress beyond that of daily life. At the time of research conception, the institution did not have a local ethical board for the fields of psychology and social sciences, and the activities were considered exempt from approval, as no identifiable data were collected. Considering the minimal risks, as well as national and international guidelines^52,53^, we considered it appropriate for the protection of participants to have them sign a proper informed consent.

### Device and soundset

An Android mobile phone was used to perform the voice labeling experiment, running a custom app implementing the VTP space described in the previous section. Participants wore Philips SHL3160RD closed-back headphones at a comfortable sound level, kept constant for all stimuli and all participants. The interaction occurred through the touchscreen, as in normal mobile use. The sound space of the Faust implementation of FOF synthesis was sampled as follows:

- *Vowel* the 25 vowels, represented as numbers from 0 to 4.8 in steps of 0.2, are obtained as interpolations of the five cardinal vowels /a, e, i, u, o/, represented as integers from 0 to 4;
- *Pitch* the range from $80~{\textrm{Hz}}$ to $1000~{\textrm{Hz}}$ (3.64 octaves) is discretized into 9 levels according to the geometric sequence of frequencies $f_i = 80 \left( \frac{1000}{80}\right) ^\frac{i}{8}$, $i = \left[ 0\mathrel {{.}\,{.}}9\right]$;
- *Type* the voice types are labeled as Bass, Tenor, Countertenor, Alto, and Soprano, in this order.

In particular, for a given voice type, the parameters of the five formants characterizing two neighboring vowels in the sequence (e.g., /i, u/) are linearly interpolated to obtain the four intermediate vowels. In total, the discretized FOF sound space is made of $9 \times 5 \times 5 = 1125$ samples. All samples are produced in real-time by the FOF synthesizer, with an overall gain set to 0.5, the vibrato (or frequency modulation) rate set to $6~{\textrm{Hz}}$, and the vibrato gain set to 0.5.

### Participants and procedure

Twentyfive Italian volunteers (13 female) with ages ranging from 19 to 62 (mean 35.64, standard deviation 15.10) participated in the experiment, in a quiet environment. Of the pool of participants, 16 were under forty years old (9 female) and 9 were over forty years old (4 female). Three participants actively play a musical instrument and two have significant experience in singing, but we have not been seeking musical expertise any further, as the target users of vowel-based auditory displays are not the music-educated analytical listeners.

To avoid the perception of a virtual source in the middle of the head, as with diotic listening, the audio was played monoaurally from one channel of the headphones: 12 participants used the right ear, 13 participants used the left ear.

Participants were briefed about the purpose of the study and asked to fill out and sign an informed consent form. Then, they were introduced to the task and given the mobile phone running the app. Each participant was requested to enter their age and gender, and underwent a short training session, accessible through a button of the mobile app, requiring the labeling of five test vowel sounds. The actual task consisted of labeling, with a five-alternative forced choice, each of 45 sounds, randomly and uniquely chosen for each participant from the set of 1125. After hearing each vowel, the participant was expected to select one among five software buttons, corresponding to the five choices /a, e, i, o, u/, visually displayed in this alphabetical order, to communicate the vowel closest to the stimulus just heard.

Overall, the purpose of the procedure was to get the full labeling of the 1125 cells of the cylindrical volume of the VTP space, by randomly assigning the cells to the pool of participants. Neighboring cells will then be aggregated to extract information at a coarser grain of analysis of the space.

**Figure 3:**
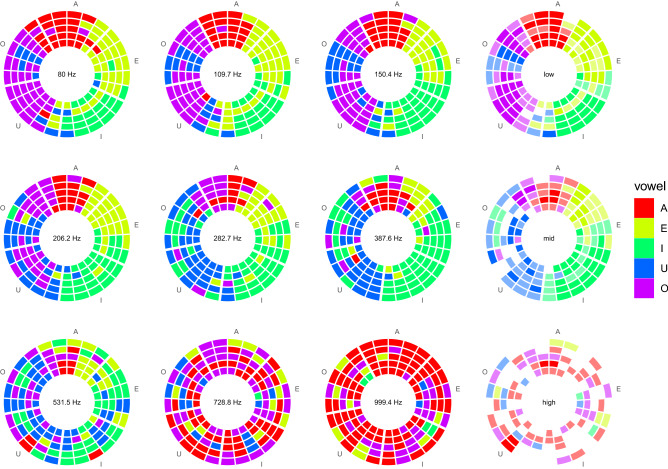
Result of the vowel labeling experiment of the VTP space, discretized into 1125 cells, each cell being labeled by one participant. The five concentric rings represent the five voice types, from bass (inner ring) to soprano (outer ring). The vowels are arranged along the circular dimension. Three leftmost columns: Reported vowels on nine slices of the VTP space, for pitches set to $80.0, 109.7, 150.4, 206.2, 282.7, 387.6, 531.5, 728.8, 999.4~{\textrm{Hz}}$; Rightmost column: Prevalent reported vowels for low (80–150.4 Hz), medium (206.2–387.6 Hz), and high (531.5–999.4 Hz) pitches, full saturation representing perfect agreement, and white representing maximal disagreement.

## Results

The responses of all participants have been color-coded with a five-step rainbow colormap and positioned in the cylindrical grid of the VTP space, displayed in Fig. 3. Each cell position corresponds to a synthesized vowel, and its color corresponds to the forced-choice response given by one participant. The nine slices corresponding to the nine pitch levels are displayed in the three leftmost columns of Fig. 3. The rainbow (pseudospectral) colormap gives maximal visual discriminability^1^ to the five vowel categories and, by sampling a closed path in the chromaticity diagram, emphasizes the circularity of vowels in the formant plane (Fig. 1). In the rightmost column of Fig. 3, the results are grouped and summarized into three pitch ranges: (1) low (80–150.4 Hz), (2) medium (206.2–387.6 Hz), and (3) high (531.5–999.4 Hz). In each frequency band, each cell of the disk contains the color code of the vowel chosen for the majority of the three pitches, with full saturation where all three reported vowels are the same, low saturation (50% transparency) where there is a two-over-three majority, and a blank cell where all three sounds are reported as different vowels.

This representation gives an immediate view of the consistency in vowel reporting across voice types and pitches, and it can guide the carving of a sound gamut in the VTP space. Given the limits of the discretization steps in the three dimensions and of the individual assignment of every single cell, the visualization of the grid of 1125 responses by 25 participants enables qualitative analysis of the proposed sound space.

To derive an index of accuracy of vowel identification, the values of the vowel parameter of the FOF synthesizer have been aggregated into neighborhoods of each nominal cardinal vowel, as $A = \{4.6, 4.8, \mathbf{0.0}, 0.2, 0.4\}$, $E = \{0.6, 0.8, \mathbf{1.0}, 1.2, 1.4\}$, $I = \{1.6, 1.8, \mathbf{2.0}, 2.2, 2.4\}$, $U = \{2.6, 2.8, \mathbf{3.0}, 3.2, 3.4\}$, $O = \{3.6, 3.8, \mathbf{4.0}, 4.2, 4.4\}$. In each set, the parameter value producing the nominal synthetic vowel is highlighted, and the other values are used for linear interpolation between neighboring cardinal vowels. Given a vowel parameter value, the response is labeled as accurate if it corresponds to the name of the set containing that value. Table 1 shows the contingency table for the collected responses of all 1125 synthetic vowel stimuli, with each row counting all synthetic vowels produced for a given set of parameters values, for all five voice types and all nine pitches. Perfect accuracy would be obtained with a table having non-null elements only along the diagonal, with a value of 225 (one fifth of 1125). On the other hand, a uniformly random distribution of responses would give a table where all elements have a value of 45, or $20\%$ correct guesses. That there is a significant association between synthetic vowel sets and reported vowels is confirmed by the chi-square test ($\chi ^2 = 763.55$, $\mathrm{d.f.} = 16$, $p < .001$). The main diagonal of Table 1, translated to percentages of correct guess, gives the values: $A: 53\%,~E: 45\%,~I: 59\%,~U: 33\%,~O: 38\%$.

**Table 1:** Contingency table for vowel identification of all 1125 stimuli.

		Reported				
		A	E	I	U	O
Generated	A	$\boxed {120}$	50	4	1	50
	E	21	$\boxed {102}$	68	12	22
	I	28	24	$\boxed {132}$	22	19
	U	35	15	15	$\boxed {75}$	85
	O	28	7	20	85	$\boxed {85}$

Each row corresponds to a set of five synthetic vowels generated around a nominal value of the vowel parameter. Each column corresponds to one of the five possible reported vowels.

**Figure 4:**
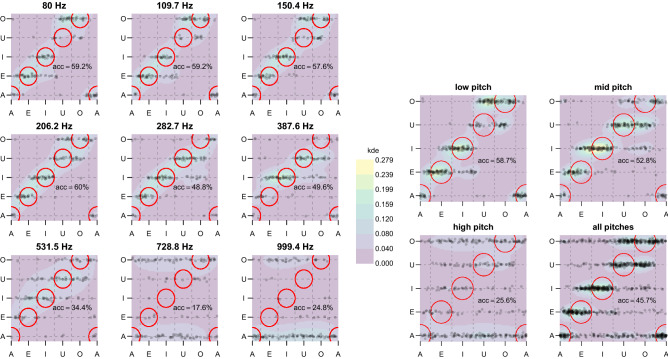
Vowel labeling accuracy (abbreviated acc). In each scatterplot, the horizontal axis represents the synthetic vowel set and the vertical axis is quantized to the five reported vowels. A zero-mean 0.05-std gaussian jitter has been added, and individual responses have been rendered as tiny black dots with 80% transparency. The background shows a bivariate kernel density estimate. The red circles represent the areas where most points are expected for accurate labeling. (Left) Vowel labeling accuracy at the nine different pitches; (Right) Vowel labeling accuracy in the three pitch ranges and for all pitches.

To better understand how each synthetic vowel set is mapped to the reported vowel labels at the different pitches, a sequence of scatterplots is reported in Fig. 4 (left). In each scatterplot, the horizontal axis represents the synthetic vowel set and the vertical axis is quantized to the five reported vowels. To reduce visual overlap, a zero-mean 0.05-std gaussian jitter has been added, and individual responses have been rendered as tiny black dots with 80% transparency. The background has been colored with a 7-values Viridis colormap to represent a bivariate kernel density estimate on a grid of  $151 \times 151$ points, as computed by the kde function of the R package ks, with default parameters. Red circles highlight the areas where a denser distribution of points is expected in case of good matching between generated and perceived vowels. Each individual scatterplot reports the measured accuracy at the corresponding pitch, that is the number of correct labels divided by the total number of stimuli, aggregated on vowels, and expressed as a percentage. An aggregation of results in the three ranges of the low, medium, and high pitch, with the corresponding accuracy values, is reported in Fig. 4 (right). The overall accuracy is $45.7\%$. Vowels are reported more accurately for low ($58.7\%$) and medium ($52.8\%$) pitches, and the classification performance is severely degraded at high pitches ($25.6\%$). In another study, using the alphabetically-ordered and interpolated /a, e, i, o, u/ sequence (supplementary material), the overall accuracy turned out to be $41.6\%$, and the values of accuracy for low, medium, and high pitches were $59.7\%$, $45.1\%$, and $20\%$, respectively.

**Figure 5:**
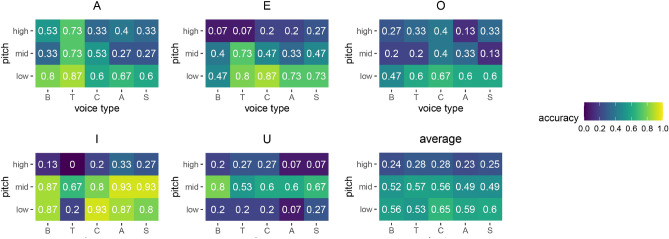
Labeling accuracy of the five vowel sets for different voice types: Bass (B), Tenor (T), Countertenor (C), Alto (A), Soprano (S). In the lower right corner, the accuracy is averaged across vowels.

The behavior of different synthetic voice types in terms of per-vowel labeling accuracy is reported in Fig. 5, and shows the better recognizability of vowels /a, e, i/ across voice types, with possible different behavior of the tenor type. Figure 5 also shows how the overall accuracy does not vary much across voice types. Accuracy is higher in the low-pitch range where it varies between 0.53 for tenor to 0.65 for countertenor.

## Discussion

Looking at the summary charts in the rightmost column of Fig. 3, we notice that the consistency in vowel labeling is relatively high at low pitches and very low for high pitches, where a large number of cells is white, thus meaning the lowest agreement between participants for the same vowel in the same pitch range. Figure 4 confirms that to be able to name at least four vowels consistently, the sound space should be limited in pitch to less than three octaves if the lowest pitch is set at $80~{\textrm{Hz}}$. Beyond that limit, the diagonal ridge in the kernel density estimate completely disappears and the reported vowels are in large majority /a/ and /o/, with a clear prevalence of the former at very high pitches. This is consistent to what is observed in listening experiments with isolated high-pitched sung vowels produced by real singers^30^. The accuracy is quite stable around $60\%$ for less than two octaves, at least from 80 Hz to 206.2Hz, and drops dramatically beyond the third octave. The upper limit of decent labeling of vowels coincides with the lower end of the pitch range for soprano singers. In fact, the fundamental frequency of a soprano vocalization is typically above the first formant frequency, which can be raised together with high values of pitch^27^. This means that the first formant frequency becomes pitch-dependent in real soprano singing while being pitch-independent in the used FOF synthesis model.

A relevant finding is that /u/ and /o/ tend to be misidentified at those pitches where the other vowels get more robustly identified: There is a prevalence of reported /o/ at low pitches, and /u/ gets reported more often at medium pitches, as it can be readily seen in Fig. 5. These two vowels share the lower left quadrant in the plane of the lowest two formant frequencies (Fig. 1). Another study, using the /a, e, i, o, u/ sequence (supplementary material), showed similar results of /o/ and /u/ misidentification and inversion at low pitches. The more natural circular interpolation in the plane of Fig. 1, given by the sequence /a, e, i, u, o/, while showing a modest improvement in overall accuracy (from $41.6\%$ to $45.7\%$), does not seem to introduce relevant benefits to perceptually separate /u/ from /o/.

The average labeling accuracy values displayed in Fig. 5 show that the five voice types behave quite consistently in each of the three pitch ranges, with an accuracy between $53\%$ and $65\%$ at a low pitch, and accuracy between $23\%$ and $28\%$ at a high pitch. The voice type that shows the highest values of vowel labeling accuracy in the low-pitch range is the countertenor, while the tenor is the least accurate. It is worth noticing that random guessing would give an accuracy of $20\%$. It is also important to stress that the number of categories of reliable vowel categorization is generally much lower than the number of discriminable vowels, as several perceptually different sounds, for a given voice type and pitch, may be given the same label.

The observation, made after Fig. 5, that average accuracy does not vary much across voice types, implies that the participants did not have particular difficulty in assigning one out of five labels to a voice playing much lower or much higher than its natural range such as, for example, to a soprano voice playing very low pitches. This confirms that the sound synthesis space, whose compact and convex structure overcomes the limitations and idiosyncrasies of real voices, is interpretable to its full extent.

The reported accuracy values certainly suffer from the division of the circular slice of the VTP cylinder into five equally-wide sectors, where the angular width corresponds to a unit step in the FOF-synthesizer vowel parameter. Indeed, the circles of Fig. 3 show that the areas of consensus around the different vowel labels can vary in width, position, and uniformity. For example, at low pitch about one-third of the ring space is labeled as /u/, and the /o/ area is much compressed, thus indicating a non-linear warping of the vowel space. The fact that naming the samples of a surface area gives very uneven patches is also well known for color spaces^54,55^. Based on the results of the vowel labeling experiment, a compensation for expansion or contraction of certain areas may be introduced at the level of interpolation of the synthesizer parameters. Still, if the purpose is that of reliably identifying the five vowels at each pitch level and for different voice types, Fig. 3 indicates areas that serve such purpose, for most types in the low and medium pitch ranges. Therefore, trajectories in the synthesizer parameter space can be drawn for each identifiable vowel across voice type and pitch.

The results of the labeling experiment give *a posteriori* justification to some of the choices that were introduced in previous successful sonifications based on voice synthesis. In particular, for the auditory display of mathematical functions, the segment /a, e, i/ of the formant space was chosen to construct a bipolar scale for the first derivative, and the pitch was limited to the octave 110–220 Hz to display function values^15^. These ranges are within the areas of the largest labeling accuracy in the VTP space.

The measured values of vowel recognition accuracy are compatible with the results obtained with real singers when their vowels are isolated^28–30^. In both everyday and musical listening, linguistic context and coarticulation play a major role at improving word intelligibility and, therefore, correct vowel identification. This is likely to happen in dynamic information sonification as well, as previous demonstrations of vocal sonification have convincingly shown^14^. Comparing the vowel recognition accuracy in the VTP space with other target-identification experiments from the sonification literature is difficult, as the task is generally different. We may limit the attention to static and passive listening settings and notice, for example, that an overall 41% of correct answers was reported for the choice of one target among 16, represented with two-dimensional psychoacoustic sonification^56^. To compare psychoacoustic to voice-based sonification a similar experiment should be run where a $4 \times 4$ matrix of vowels and pitches is mapped to sixteen targets, and participants are previously exposed to the association.

A limitation of this study is that the discretized VTP space contains only one label for each sample. Statistics are not available for each sample and are only aggregated across collections of samples, based on pitch range or voice type. For a finer description of the space, future studies should examine only sectors that are consistently labeled. It should be noted that the entire VTP space is usable, and even the portions of the pitch-type plane where only two vowel labels are reported can be used in sonification since continuous timbral paths can be constructed between them^17^. In this study, no confidence level is associated with the labeling of each individual sample in the VTP space. For such measures, a much broader set of measurements would need to be collected. The interpretation of the study results is supported by the fact that they are consistent with prior research and with a preliminary study (see Appendix) based on a strictly alphabetical sequence of vowels (/o/ and /u/ are switched in the main study). This study has not attempted to label the voice types, as it is difficult to do so with isolated vowels, even if they come from real singers. However, the fact that different types are difficult to label does not mean that they can not be discriminated. Order and discriminability can be enforced by adding a filter-based brightness control, thus making the VTP model essentially a specialization of the TBP model^7^. To avoid the introduction of a confounding variable in the vowel labeling test, we avoided using a brightness filter in our study, which covered only FOF synthesis with no extensions. Future research could validate the brightness-adjusted type dimension.

As an answer to the RQ emerging from the VTP implementation and testing, a few guidelines can be drawn for the design of a perceptual sound space for auditory display and sonification, that can render multivariable data described along nominal, ordinal, and interval/ratio scales. FOF synthesis of sung vowels is suitable as a sound engine for real-time continuous and perceptually-consistent navigation of a sound space, as long as:

- Vowels are circularly interpolated in the order /a, e, i, u, o/, with a remapping of the vowel parameter for /u/ and /o/ in the low-pitch range;
- Voice types are presented in the order: Bass, Tenor, Countertenor, Alto, Soprano. The voice type axis can be made continuous by formant parameter interpolation. Variations along such dimension can be emphasized if a properly-calibrated brightness filter is added, thus making low types darker and high types brighter;
- Pitch is limited to less than three octaves in the low–medium range. Extension beyond the third octave reduces the number of perceived vowel categories to two at most.

## Conclusion and outlook

We introduced the VTP three-dimensional perceptual sound space based on categorizable sung Vowels, ordinally arranged voice Types, and an interval scale of Pitches. The VTP space was mapped to the parameters of a formant-wave-function synthesizer and implemented as a mobile app for testing how consistent the labeling of vowels is across different types and pitches. Results from a vowel labeling experiment showed that effective categorization and labeling are possible at low–medium pitches, whereas higher fundamental frequencies lead to the dominance of categories /a/ and /o/.

The results have implications for the design of auditory and multi-sensory displays that account for human perceptual capabilities and afford embodied cognitive modeling and interaction. In information sonification, data streams can be associated with distinct voices that can be individually followed as their pitch varies, and reliably named at each time instant. For example, to focus on a specific data subset, the analyzer just steers the attention toward a specific vowel being sung by a tenor voice, thus perceptualizing data points as if they were notes of a vocal counterpoint. In a future experiment, the orbit-synchronization object selection paradigm^57^, where one of a few orbiting displays is selected by synchronized motion gesture, will be adopted and tested in the audio domain. Here, a few streams, each associated with a voice type, orbit circularly in a space of vowel-pitch, spanning a range of pitches and interpolating between two vowels. Stream selection may be done by synchronizing a circular gesture or by vocal imitation of one of the streams.

The VTP model can be extended by looking at other kinds of vocal emissions, and modeling turbulent or supraglottal excitation. If the periodic excitation (corresponding to phonation) of the formant resonances is replaced with a noise source (as in turbulent excitation), we can add a pitchless disc to the VTP cylinder, thus effectively extending the sound volume with a subspace that is perceptually distinct.

If all three dimensions are made to vary continuously, sound can be synthesized along a continuous trajectory in the VTP cylinder. On the other hand, discrete positions in the VTP space can be made easier to distinguish by adding consonant-like transients^27,28,41^. A hyperspace may be constructed as a collection of VTP cylinders, each corresponding to a consonant and a set of syllables (e.g., /ta, te, ti, tu, to/), thus adding another categorical dimension. This would also improve the accuracy of vowel labeling, as it has long been known that, under most circumstances, listeners identify vowels in consonant contexts more accurately than vowels in isolation^28^.

The proposed sound space affords continuous sonic interaction by variation along all of its three dimensions. As an important application, the sonification of continuous body gestures may be transformed into vocal gestures that are perceived as signatures of motion patterns in multisensory biofeedback systems^17^. Such correspondence can be actually reversed so that human vocalizations can be used to specify and control trajectories and motion patterns in sonic interactions^58^.

We expect to see more examples and studies of vowel-based sonification of data, events, and processes, possibly with a comparison with other conceptual metaphors and sonification methods in terms of aesthetics, engagement, and specific task performance.

## Supplementary Information

Supplementary Information 1.

Supplementary Information 2.

Supplementary Information 3.

## Data Availability

All data generated or analyzed during this study are included in this published article (and its Supplementary Information files).
