# Supplementary material for: A perceptual sound space for auditory displays based on sung-vowel synthesis
Source: Sci Rep. 2022 Nov 12;12:19370. doi: 10.1038/s41598-022-23736-2 (PMC9653387; doi:10.1038/s41598-022-23736-2)
Supplement: Supplementary file 2 — Supplementary Information 2. [file 41598_2022_23736_MOESM2_ESM.pdf]

Appendix to "A perceptual sound space for auditory displays based on sung-vowel synthesis"

Davide Rocchesso[1], Salvatore Andolina[1,*], Giacomo Ilardo[1], Salvatore Danilo Palumbo[1], Ylenia Galluzzo[1], and Mario Randazzo[1]

[1]University of Palermo, Department of Mathematics and Computer Science, Palermo, 90123, Italy
[*]salvatore.andolina@unipa.it

Preliminary study

In this section we report a preliminary study conducted to get a first understanding of whether humans could unambiguously assign labels (vowel names) to different sectors of the VTP cylindrical sound space, and how robust such labeling would be across voice types and in different pitch ranges. The goals, procedure and analysis of results are the same as the main study. Similar to what done in the main study, for the categorical dimension of vowels, we used the five cardinal vowels. However, in this preliminary study, instead of modifying the synthesis engine, we chose to use the default /a, e, i, o, u/ sequence that is implemented in the FOF synthesizer as a continuous parametric path, with interpolation between neighboring vowels.

Participants and procedure

Twenty-five volunteers (14 female, 11 male) with ages ranging from 18 to 61 (mean 36.48, standard deviation 13.35) participated in the experiment, in a quiet environment. Of the pool of participants, 13 were under forty years old (6 female, 7 male) and 12 were over forty years old (8 female, 4 male). Audio was played monoaurally from one channel of the headphones: 12 participants used the right ear, 13 participants used the left ear.

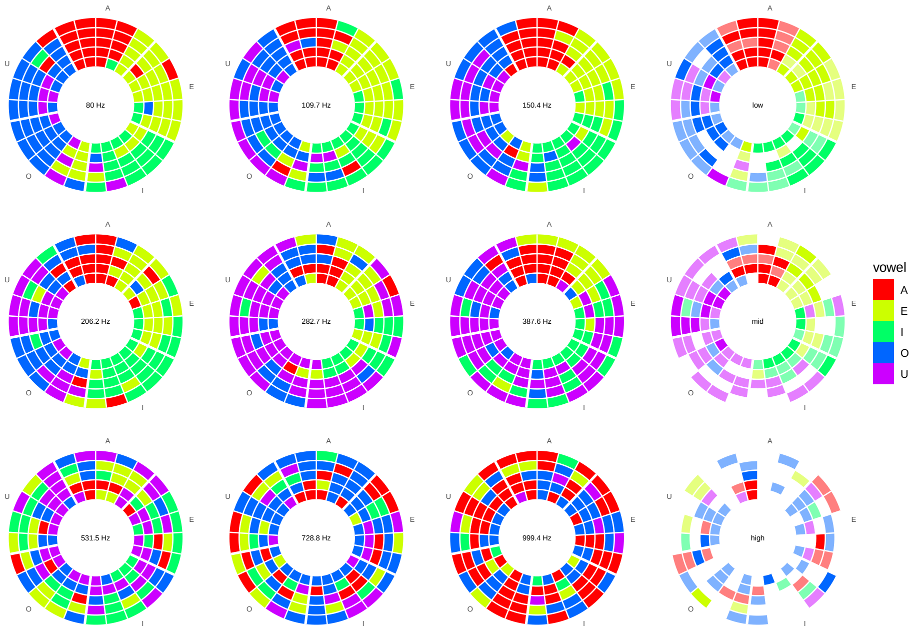

Figure S1. Result of the vowel labeling experiment. Left: Reported vowels on nine slices of the VTP space, for pitches set to 80.0, 109.7, 150.4, 206.2, 282.7, 387.6, 531.5, 728.8, 999.4Hz; Right: Prevalent reported vowels for low (80 – Hz), medium (206.2 – Hz), and high (531.5 – Hz) pitches, full saturation representing perfect agreement, and white representing maximal disagreement.

		Reported				
		A	E	I	O	U
Generated	A	114	49	7	13	42
	E	23	94	47	31	30
	I	16	24	107	43	35
	O	26	30	18	64	87
	U	22	14	13	87	89

Table S1. Contingency table for vowel identification of all 1125 stimuli. Each row corresponds to a set of five synthetic vowels generated around a nominal value of the vowel parameter. Each column corresponds to one of the five possible reported vowels.

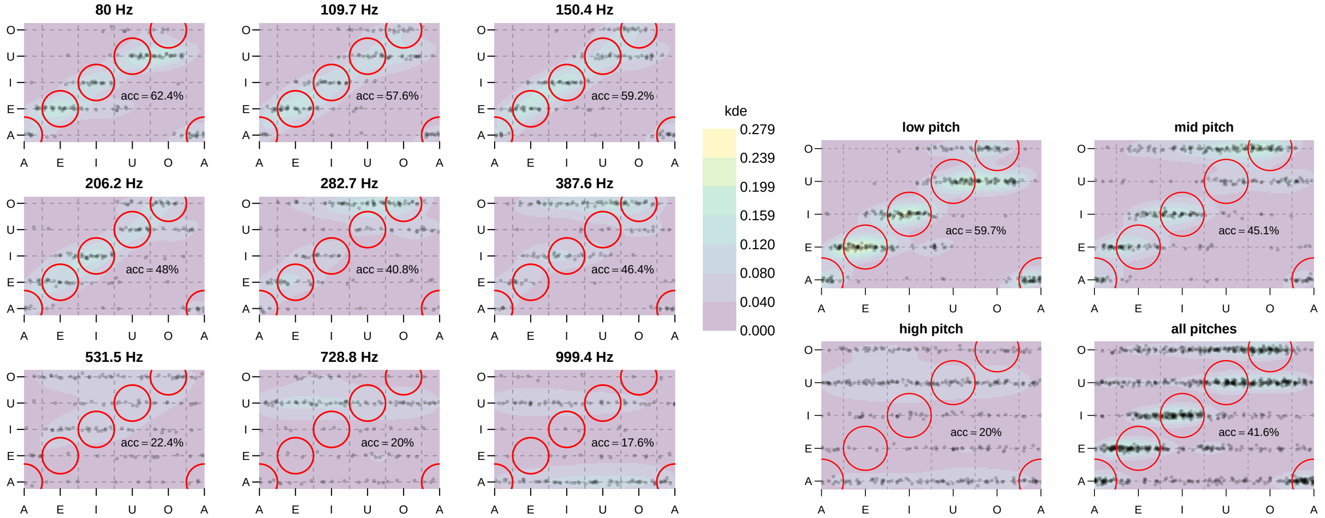

Figure S2. Vowel labeling accuracy (abbreviated `acc`). In each scatterplot, the horizontal axis represents the synthetic vowel set and the vertical axis is quantized to the five reported vowels. A zero-mean 0.05-std gaussian jitter has been added, and individual responses have been rendered as tiny black dots with 80% transparency. The background shows a bivariate kernel density estimate. The red circles represent the areas where most points are expected for accurate labeling. (Left) Vowel labeling accuracy at the nine different pitches; (Right) Vowel labeling accuracy in the three pitch ranges and for all pitches.

Results

The results of this preliminary experiment are shown in Figure S1. The responses of all participants have been color-coded with a five-step rainbow colormap and positioned in the cylindrical grid of the VTP space, with the same criteria used in the main study. Table S1 shows the contingency table for the collected responses of all 1125 synthetic vowel stimuli, with each row counting all synthetic vowels produced for a given set of parameters values, for all five voice types and all nine pitches. Perfect accuracy would be obtained with a table having only non-null elements along the diagonal, with value 225. On the other hand, a uniformly random distribution of responses would give a table where all elements have value 45. That there is a significant association between synthetic vowel sets and reported vowels is confirmed by the chi-square test ($\chi^2 = 572.39$, d.f. $= 16$, $p < .001$).

To better understand how each synthetic vowel set is mapped to the reported vowel labels at the different pitches, a sequence of scatterplots is reported in Fig. S2 (left). In each scatterplot, the horizontal axis represents the synthetic vowel sets and the vertical axis is quantized to the five reported vowels. To reduce visual overlap, a zero-mean 0.05-std gaussian jitter has been added, and points have been rendered with 80% transparency. The background has been colored with a 7-values Viridis colormap to represent a bivariate kernel density estimate. Red circles highlight the areas where a denser distribution of points is expected in case of good matching between generated and perceived vowels. Each individual scatterplot reports the measured accuracy at the corresponding pitch. An aggregation of results in the three ranges of low, medium, and high pitch, with the corresponding accuracy values, is reported in Fig. S2 (right). The overall accuracy is 41.6%. Vowels are reported more accurately for low (59.7%) and medium (45.1%) pitches, and the classification performance is severely degraded at high pitches (20%). In the follow-up study (section IV), where we tweaked the synthesis engine to use the more suitable /a, e, i, u, o/ sequence describing a closed triangular trajectory in the formant plane, the overall accuracy turned out to be 45.7%, and the values of accuracy for low, medium and high pitches were 58.7%, 52.8%, and 25.6%, respectively. The behavior of different synthetic voice types in terms of per-vowel labeling accuracy is reported in Fig. S3.

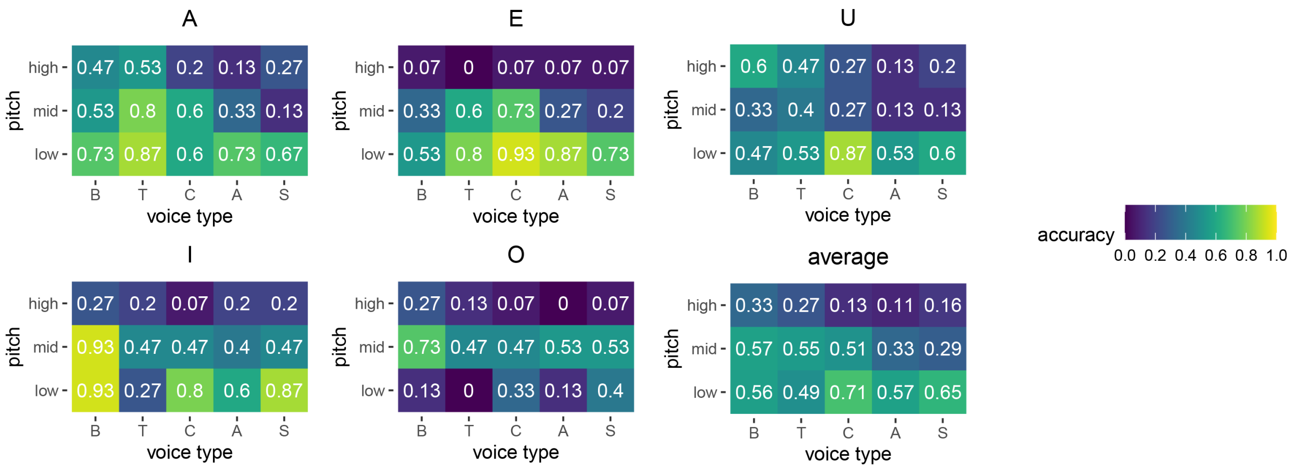

Figure S3. Labeling accuracy of the five vowel sets for different voice types: Bass, Tenor, Countertenor, Alto, Soprano.
