# Supplementary figures and images for: A perceptual sound space for auditory displays based on sung-vowel synthesis
Source: Sci Rep. 2022 Nov 12;12:19370. doi: 10.1038/s41598-022-23736-2 (PMC9653387; doi:10.1038/s41598-022-23736-2)

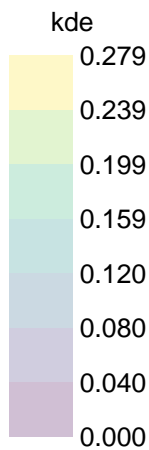

Supplement: Supplementary file 3 — Supplementary Information 3. [file 41598_2022_23736_MOESM3_ESM.zip › Supplementary Materials - Preliminary study/campione/legendScatterplots (1).pdf]

**low pitch**

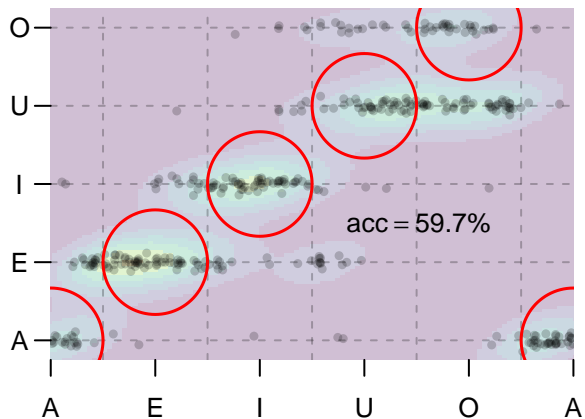

**mid pitch**

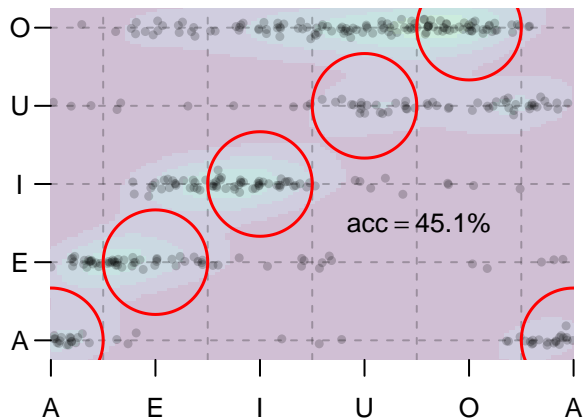

**high pitch**

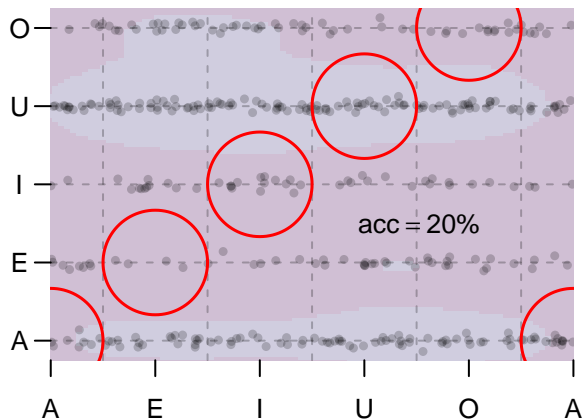

**all pitches**

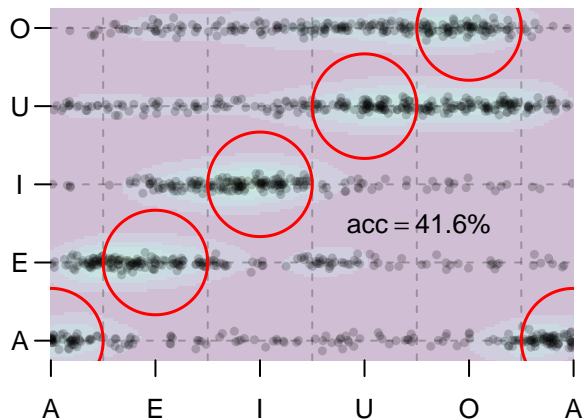

Supplement: Supplementary file 3 — Supplementary Information 3. [file 41598_2022_23736_MOESM3_ESM.zip › Supplementary Materials - Preliminary study/campione/Rplot.pdf]

**low pitch**

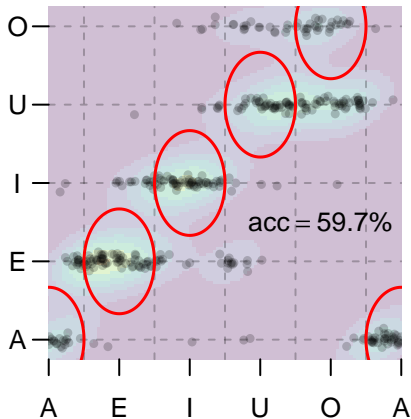

**mid pitch**

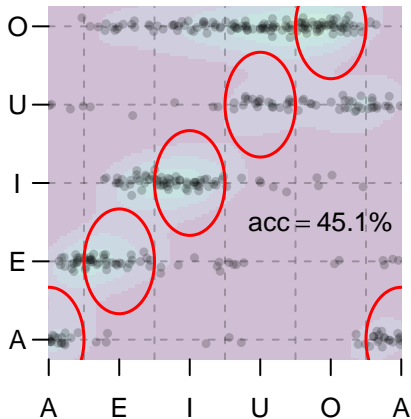

**high pitch**

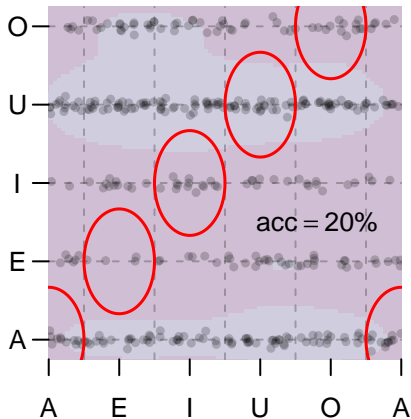

**all pitches**

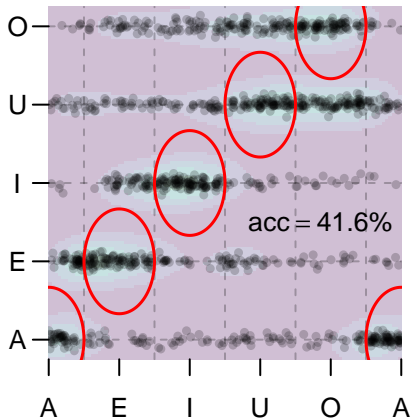

Supplement: Supplementary file 3 — Supplementary Information 3. [file 41598_2022_23736_MOESM3_ESM.zip › Supplementary Materials - Preliminary study/campione/Rplot01.1.pdf]

**80 Hz**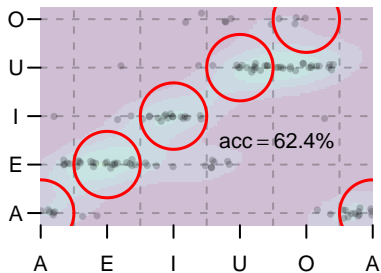**109.7 Hz**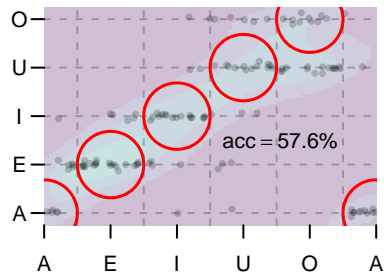**150.4 Hz**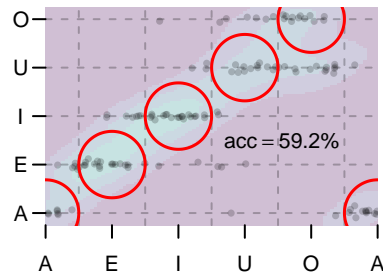**206.2 Hz**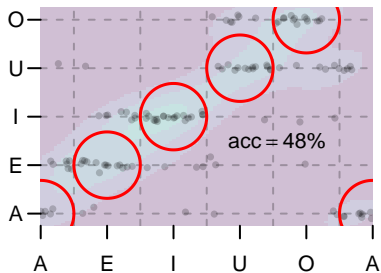**282.7 Hz**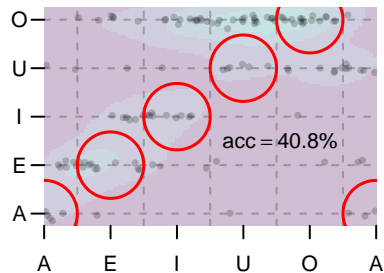**387.6 Hz**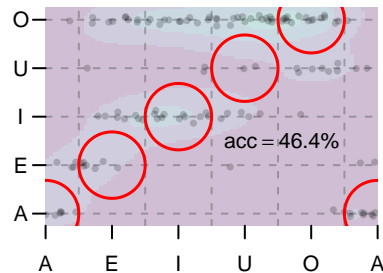**531.5 Hz**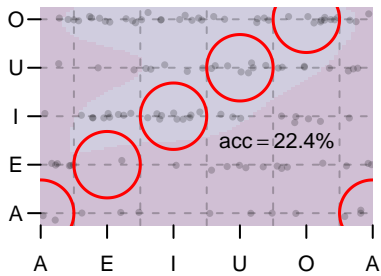**728.8 Hz**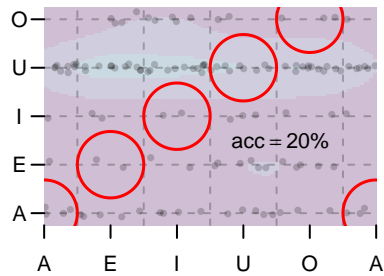**999.4 Hz**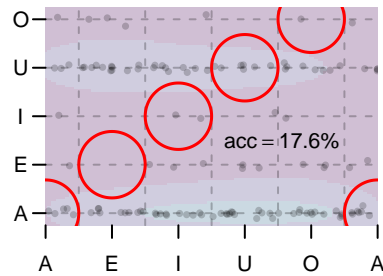

Supplement: Supplementary file 3 — Supplementary Information 3. [file 41598_2022_23736_MOESM3_ESM.zip › Supplementary Materials - Preliminary study/campione/Rplot02.pdf]

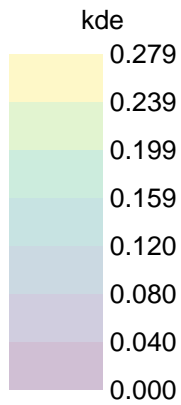

Supplement: Supplementary file 3 — Supplementary Information 3. [file 41598_2022_23736_MOESM3_ESM.zip › Supplementary Materials - Preliminary study/campione/Rplot03.pdf]
